# Duodenal Rare Neuroendocrine Tumor: Clinicopathological Characteristics of Patients with Gangliocytic Paraganglioma

**DOI:** 10.1155/2016/5257312

**Published:** 2016-12-21

**Authors:** Yoichiro Okubo, Tomoyuki Yokose, Osamu Motohashi, Yohei Miyagi, Emi Yoshioka, Masaki Suzuki, Kota Washimi, Kae Kawachi, Madoka Nito, Tetsuo Nemoto, Kazutoshi Shibuya, Yoichi Kameda

**Affiliations:** ^1^Department of Pathology, Kanagawa Cancer Center, 2-3-2 Nakao, Asahi-Ku, Yokohama, Kanagawa 241-8515, Japan; ^2^Department of Gastroenterology, Kanagawa Cancer Center, 2-3-2 Nakao, Asahi-Ku, Yokohama, Kanagawa 241-8515, Japan; ^3^Molecular Pathology and Genetics Division, Kanagawa Cancer Center Research Institute, 2-3-2 Nakao, Asahi-Ku, Yokohama, Kanagawa 241-8515, Japan; ^4^Department of Thoracic Surgery, Kanagawa Cancer Center, 2-3-2 Nakao, Asahi-Ku, Yokohama, Kanagawa 241-8515, Japan; ^5^Department of Surgical Pathology, Toho University School of Medicine, 6-11-1 Omori-Nishi, Ota-Ku, Tokyo 143-8541, Japan

## Abstract

Gangliocytic paraganglioma (GP) has been regarded as a rare benign tumor that commonly arises from the second part of the duodenum. As GP does not exhibit either prominent mitotic activity or Ki-67 immunoreactivity, it is often misdiagnosed as neuroendocrine tumor (NET) G1. However, the prognosis might be better in patients with GP than in those with NET G1. Therefore, it is important to differentiate GP from NET G1. Moreover, our previous study indicated that GP accounts for a substantial, constant percentage of duodenal NETs. In the present article, we describe up-to-date data on the clinicopathological characteristics of GP and on the immunohistochemical findings that can help differentiate GP from NET G1, as largely revealed in our new and larger literature survey and recent multi-institutional retrospective study. Furthermore, we would like to refer to differential diagnosis and clinical management of this tumor and provide intriguing information about the risk factors for lymph node metastasis on GP.

## 1. Introduction 

The incidence of neuroendocrine tumors (NETs) has been increasing worldwide [1]; however, the reasons for this increase are unclear [1]. The current World Health Organization classification proposed a grading system for NETs based on the proliferative activity of tumor cells (the number of mitoses or the Ki-67 labeling index) [2–4]. Specifically, NETs have been classified as G1, G2, and G3 (neuroendocrine carcinoma). Patients with NET G1 have a relatively good prognosis; however, it has been reported that the 5-year survival rate of patients with NET G1 was approximately 80–90% [5]. We would like to emphasize that clinicians and pathologist should be aware of the existence of gangliocytic paraganglioma (GP), which is often misdiagnosed as NET G1 [6]. GP has been regarded as an extremely rare NET [7]. Dahl et al. [8] first reported this tumor as ganglioneuroma in 1957, and Kepes and Zacharias [9] named this tumor “gangliocytic paraganglioma” in 1971. Previous studies [7, 10] have reported on the clinicopathological characteristics of GP. These studies stated that this rare tumor typically occurs in the second part of the duodenum, includes 3 characteristic components (epithelioid, spindle-shaped, and ganglion-like cells), and shows a good prognosis. On the other hand, few cases with lymph node and/or liver metastasis [11–13] and 1 fatal duodenal GP case after distant metastasis (pelvic lesion and liver mass) [11] have been reported. As most previous studies included a single patient or a small group of patients, we previously evaluated the details of this tumor in accordance with the Preferred Reporting Items for Systematic Reviews and Meta-Analyses (PRISMA) style [14] as much as possible and elucidated some aspects of the epidemiology and clinicopathological characteristics of GP. Thereafter, we perused studies on GP and obtained new knowledge through a multi-institutional retrospective study of GP [6]. We found that the prognosis might be better in patients with GP than in those with NET G1, and we believe that it is important to differentiate between GP and NET G1. In this review article, we describe up-to-date data of the clinicopathological characteristics of GP, based on a new and larger literature survey and the results of our recent retrospective study [6].

## 2. Benefits of Conducting a Literature Survey

Our previous multi-institutional retrospective study [6] shed some light on clinicopathological findings of GP. However, since GP is an extremely rare neuroendocrine tumor, a large study design is difficult and standard clinical management of this tumor has not been established. Accordingly, we would like to emphasize the benefits of literature survey. This research method can provide the researcher with up-to-date key to the deciphering of GP.

## 3. Literature Survey and Data Collection Method

In January 2016, we conducted a comprehensive literature survey using the PubMed (http://www.ncbi.nlm.nih.gov/pubmed/) and Igaku Chuo Zasshi (http://www.jamas.or.jp/; Japanese medical database) databases. Namely, we assessed English and Japanese language case reports of GP using these medical databases and search term was “gangliocytic paraganglioma.” In the resent review, no advance search systems of PubMed database were used, whereas for the Igaku Chuo Zasshi search, the “shoreihoukoku” (Japanese word for case report) option was used. We then reviewed all abstracts of selected publications to identify GP cases.

In addition, since gangliocytic paraganglioma has been reported by other names (e.g., ganglioneuroma [8], nonchromaffin paraganglioma [15, 16], and paraganglioma [17, 18]) until named “gangliocytic paraganglioma” by Kepes and Zacharias in 1971 [9] and becoming widespread, some GP cases were not identified in the first databases search. We therefore checked the references of selected publications collected by first database search. With reference to our previous research [7], we added some publications as GP and these publications met the following criteria: (1) the characteristic three components could be confirmed in the manuscript or a figure and (2) the paper was cited in other publications as a GP report.

Subsequently, we extracted and sampled the raw data from the selected publications in relation to factors, such as age, sex, site of the tumor, tumor size, medical treatment method, outcome, lymph node metastasis, depth of tumor invasion, diagnostic rate using biopsy specimens before medical treatment, clinical symptoms, and immunohistochemical findings. In this literature survey, data collection was performed in accordance with the PRISMA style [14] as much as possible. In addition, we performed appropriate statistical analyses using the extracted data. The nonparametric Mann–Whitney* U* test or *χ* ^2^ test was used for statistical analysis. All statistical analyses were performed using IBM SPSS Statistics version 20 (IBM Corp., Armonk, NY, USA). Differences were considered significant at *P* < 0.05 [19].

## 4. Overall Findings of Literature Survey of GP

We retrieved 21,581 English and 40 Japanese (total 21,621) publications by conducting a search of “gangliocytic paraganglioma” using the PubMed and Igaku Chuo Zasshi databases. Among these publications, 123 English and 30 Japanese publications were recognized and selected as case reports of GP. The remaining 21,468 publications were excluded from this survey. We then reviewed the references of all the selected publications of GP; however, no additional GP case reports were found. Consequently, 254 patients with GP were finally assessed (totally, 123 English and 30 Japanese publications reported 254 patients with GP).

**Figure 1:**
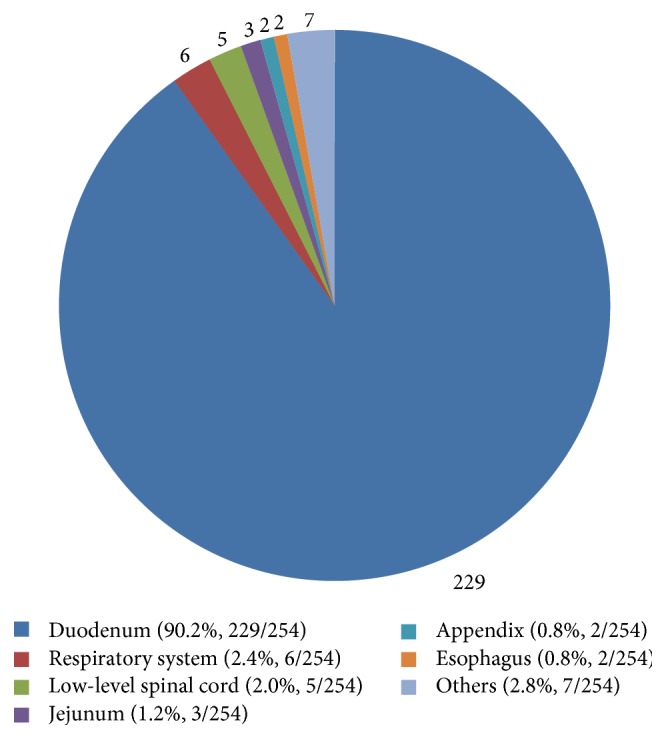
In the present literature survey, the duodenum was found to be the most common site of gangliocytic paraganglioma (90.2%, 229/254), followed by the respiratory system (2.4%, 6/254), low-level spinal cord (2.0%, 5/254), jejunum (1.2%, 3/254), esophagus (0.8%, 2/254), appendix (0.8%, 2/254), and others (2.8%, 7/254).

The patient age at the time of diagnosis ranged from 15 to 84 years (*n* = 254; mean ± standard deviation (SD), 53.22 ± 12.13). The sex ratio was 152 : 100 (male : female; *n* = 252, 2 not reported). The tumor size at the time of diagnosis ranged from 5.5 to 100 mm (*n* = 194, 60 not reported; mean ± SD, 25.73 ± 1422 mm). The duodenum was found to be the most common site of the disease (90.2%, 229/254), followed by the respiratory system (2.4%, 6/254) [20–25], low-level spinal cord (2.0%, 5/254) [26–30], jejunum (1.2%, 3/254) [31–33], esophagus (0.8%, 2/254) [34, 35], and appendix (0.8%, 2/254) [36, 37]. There were individual cases involving the stomach [31], ileum [38], retromediastinum [39], pancreas [40], thymus [41], and mature teratoma [42], as well as a case of double focus in the duodenum and pancreas [43]. These data are summarized in Figure 1. As most of the cases of GP were associated with the duodenum, we additionally conducted detailed clinicopathological examinations and statistical analyses for duodenal GP.

## 5. Clinical Findings of Duodenal GP

**Figure 2:**
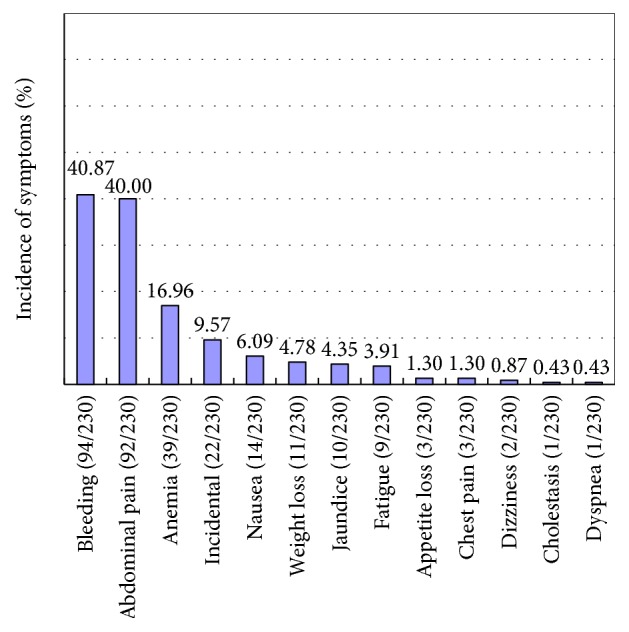
In the present literature survey, gastrointestinal bleeding was the most common symptom of gangliocytic paraganglioma (GP; 40.9%, 94/230), followed by abdominal pain (40.0%, 92/230), anemia (17.0%, 39/230), incidental findings (9.6%, 22/230), nausea (6.1%, 14/230), weight loss (4.8%, 11/230), and jaundice (4.4%, 10/230). It has been largely accepted that gastrointestinal bleeding and abdominal pain commonly occur in patients with GP; however, our survey revealed that obstructive jaundice is less common in patients with GP, although GP commonly occurs in the second part of the duodenum.

A total of 230 patients with duodenal GP were identified from the PubMed and Igaku Chuo Zasshi databases (including the case of double focus in the duodenum and pancreas [43]). The patient's age at the time of diagnosis ranged from 15 to 84 years (*n* = 230; mean ± SD, 53.60 ± 11.79). The sex ratio was 136 : 92 (male : female; *n* = 228, 2 not reported). Gastrointestinal bleeding was the most common symptom of this tumor (40.9%, 94/230), followed by abdominal pain (40.0%, 92/230), anemia (17.0%, 39/230), incidental findings (9.6%, 22/230), nausea (6.1%, 14/230), weight loss (4.8%, 11/230), and jaundice (4.4%, 10/230). These findings are summarized in Figure 2.

The documented follow-up period ranged from 3 months [44] to 300 months [45], and recurrence of GP was reported in only 2 patients [11, 46]. Unfortunately, 1 of these patients died from GP [11]. Although 26 patients underwent an endoscopic procedure for treatment, only 1 patient required additional surgical intervention owing to the presence of a tumor residue following the initial procedure [47].

## 6. Histopathological Findings of Duodenal GP

**Figure 3:**
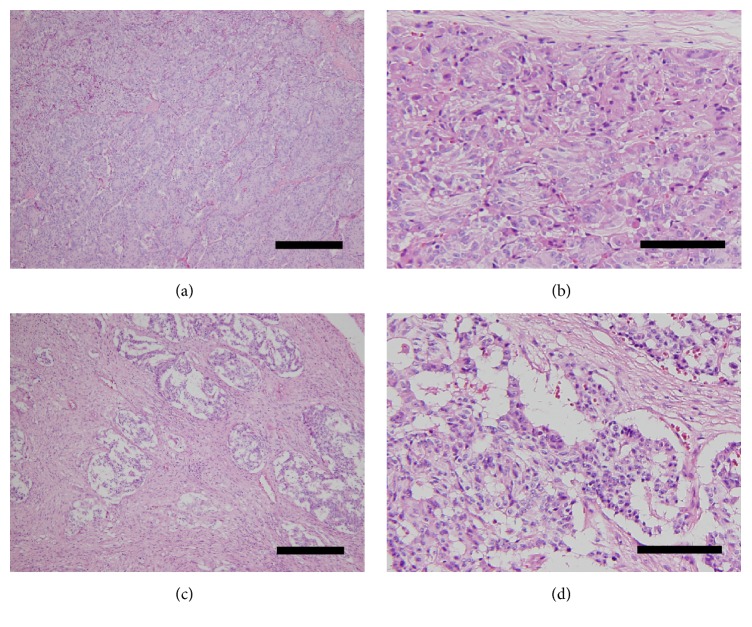
(a) Photomicrograph showing a low-power field of the gangliocytic paraganglioma (GP) site with a dense proliferation of epithelioid cells. Nested and compactly arranged epithelioid cells comprise the majority of the tumor (HE staining; magnification, ×100; the scale bar represents 300 *μ*m). (b) Photomicrograph showing a high-power field of the GP site. The epithelioid cells have round to oval-shaped nuclei, inconspicuous nucleoli, and eosinophilic cytoplasm. Spindle-shaped cells surround the nests of epithelioid cells and are aligned in a single layer (HE staining; magnification, ×400; the scale bar represents 100 *μ*m). (c) Photomicrograph showing a low-power field of the GP site with sporadic proliferation of epithelioid cells. A chaotic arrangement of epithelioid cells and a predominance of stromal cells are seen (HE staining; magnification, ×100; the scale bar represents 300 *μ*m). (d) The epithelioid cells show a random arrangement, and spindle cells in the stroma are arranged in an irregular pattern (HE staining; magnification, ×400; the scale bar represents 100 *μ*m).

In the selected cases, the tumor size at the time of diagnosis ranged from 5.5 to 100 mm (*n* = 172, 58 not reported; mean ± SD, 25.33 ± 13.41 mm). The depth of tumor invasion was described in 153 patients. Of the 153 patients, 81 had GP within the submucosa or sphincter of Oddi layer and 72 had GP exceeding the submucosa or sphincter of Oddi layer. Moreover, 25 patients showed lymph node metastasis [6, 10–13, 18, 46–60], 3 patients showed pancreatic metastasis and/or invasion [51, 55, 57], and 3 patients showed liver metastasis [11–13]. As mentioned previously, GP is an extremely rare NET, and the histopathological diagnosis of GP requires confirmation of the presence of epithelioid, spindle-shaped, and ganglion-like cells. However, the distribution of the 3 characteristic tumor cells varied from case to case, even among patients with GP [6, 61]. As histopathological findings vary widely (Figure 3), pathologists should recognize this fact when diagnosing GP. Furthermore, diagnosis using biopsy specimens obtained before surgical intervention or an endoscopic procedure has been regarded as extremely difficult in the literature. In fact, histopathological findings of biopsy specimens obtained before surgical intervention were described in 60 patients. However, among these 60 patients, 10 were successfully diagnosed with GP, 40 showed no evidence of tumor cells (specimens did not contain tumor cells), 9 were diagnosed or suspected with a different NET (6 carcinoid tumors, 2 paragangliomas, and 1 ganglioneuroma), and 1 showed atypical cells (details unknown). Therefore, in the literature, only 16.7% (10/60) of the patients with GP were accurately diagnosed using biopsy specimens.

## 7. Immunohistochemical Findings of Duodenal GP

In the present literature survey, we only assessed the cases in which the positive or negative result of the immunohistochemical analysis was clearly mentioned. Thus, the denominators for the collected immunohistochemical data varied for each kind of immunohistochemical item. The representative findings for each of the 3 characteristic tumor cells are presented below.

In epithelioid cells, CD56 showed the highest positive rate (100%, 27/27), followed by synaptophysin (96.2%, 76/79), neuron-specific enolase (NSE; 95.7%, 90/94), progesterone receptor (93.3%, 14/15), pancreatic polypeptide (91.5%, 86/94), somatostatin (82.6%, 76/92), chromogranin A (74.8%, 98/131), cytokeratins (58.8%, 50/85), vimentin (38.0%, 3/8), and estrogen receptor (23.1%, 3/13).

In spindle-shaped cells, S-100 showed the highest positive rate (96.6%, 144/149), followed by NSE (85.0%, 68/80), neurofilament (69.1%, 47/68), vimentin (60.0%, 3/5), Bcl-2 (58.3%, 7/12), synaptophysin (54.4%, 31/57), CD56 (50.0%, 9/18), CD34 (33.3%, 1/3), calcitonin (20.0%, 4/20), and vasoactive intestinal peptide (13.3%, 4/30).

In ganglion-like cells, CD56 showed the highest positive rate (100%, 19/19), followed by synaptophysin (98.4%, 61/62), NSE (87.0%, 80/92), somatostatin (51.3%, 41/80), Bcl-2 (36.4%, 4/11), pancreatic polypeptide (32.1%, 27/84), chromogranin A (28.9%, 28/97), neurofilament (27.3%, 18/66), vimentin (25.0%, 1/4), and S-100 (23.9%, 28/117). The immunohistochemical findings are summarized in Table 1.

## 8. Statistical Analysis of Clinicopathological Data 

We assessed the significant risk factors associated with lymph node metastasis and depth of invasion (indicators of tumor growth) of duodenal GP. When we used evidence of lymph node metastasis as an indicator of progression, a significant difference was found only for the tumor size among the clinicopathological findings between patients with and those without lymph node metastasis (Mann–Whitney* U* test, *P* = 0.009). The tumor size was larger in patients with lymph node metastasis than in those without metastasis. In contrast, no significant differences were found in age and sex between patients with and those without lymph node metastasis (Mann–Whitney* U* test, *P* = 0.105, and *χ* ^2^ test, *P* = 0.390, resp.). These results are summarized in Table 2.

In addition, comparisons of clinicopathological findings between patients with GP within and those with GP exceeding the submucosa or sphincter of Oddi layer revealed significant differences in sex and the rate of lymph node metastasis (*χ* ^2^ test, *P* = 0.032 and *P* = 0.019, resp.). The number of female patients and the rate of lymph node metastasis were higher among patients with GP exceeding the submucosa or sphincter of Oddi layer than among those with GP within the submucosa or sphincter of Oddi layer. These results are summarized in Table 3.

No significant differences were noted in tumor size and patient age between male and female patients. In addition, Spearman's rank correlation coefficient was calculated to assess any potential relationship between tumor size and patient age; however, no significant relationship was noted. These results are consistent with those of our previous study [7].

Tumor size and depth of invasion were significant risk factors for lymph node metastasis. In addition, as approximately half of the patients with GP (47.1%, 72/153) had lesions exceeding the submucosa or sphincter of Oddi layer, detailed imaging examinations to determine depth of invasion are important. On the other hand, although the overall mean tumor size of duodenal GP was 25.3 mm, the mean and median tumor size of duodenal GP associated with lymph node metastasis were 32.3 and 30.0 mm. Therefore, it might be necessary to perform imaging examinations for confirmation of the existence of lymph node metastasis if the tumor size is larger than 30 mm. Meanwhile, tumor size larger than 30 mm has another important clinical significance, because several recent reports indicate that endoscopic polypectomy is the treatment of choice, except in cases where the tumor is >30 mm [62–67]. We therefore conducted further statistical analysis between tumor size and rate of lymph node metastasis. In the present survey, 185 cases described both tumor size and presence or absence of lymph node metastasis. We carried out statistical examinations for confirmation of the existence of lymph node metastasis if the tumor size is larger than 30 mm. As a result, rate of lymph node metastasis in GP within 30 mm (tumor size) was 16 of 156 cases (10.3%) and the rate in GP larger than 30 mm (tumor size) was 9 of 29 cases (31.0%), respectively. In addition, significant difference was found between them (*χ* ^2^ test, *P* = 0.006). This fact indicated that GP larger than 30 mm is also significant risk factor of lymph node metastasis. This fact indicated that GP larger than 30 mm is risk factor of not only clinical complications but also lymph node metastasis. These results are summarized in Table 4.

In the present review, sex was not significantly associated with tumor size and the rate of lymph node metastasis. However, female sex was associated with GP exceeding the submucosa or sphincter of Oddi layer. This finding indicates that female patients mainly show vertical tumor growth. Epithelioid cells (typically the main component of GP) showed positive immunoreactivity for the progesterone receptor, and some investigators reported that progesterone regulates neural differentiation [68, 69], suggesting that the vertical growth of GP might be influenced by progesterone exposure. However, our survey might be affected by publication bias, because the findings were based on a cumulative case series. Therefore, further studies are required to confirm our findings. For example, analyses of gene expression using stored formalin-fixed paraffin-embedded tissue which is widely employed in routine works for surgical pathology may be necessary, as in other fields [70–72].

## 9. Differential Diagnosis of GP

The diagnosis of GP using specimens obtained during surgical removal or endoscopic resection is not always difficult, because the characteristic 3 components can be confirmed with ease. In contrast, the diagnosis using biopsy specimens has been regarded as extremely difficult. In fact, our present review found that the biopsy diagnostic rate before surgical intervention was only 16.7% (10/60) in the literature. The differential diagnoses include gastrointestinal stromal tumor, smooth muscle tumor, NET, ganglioneuroma, and paraganglioma. Gastrointestinal stromal tumor, smooth muscle tumor, and adenocarcinoma can easily be excluded with immunohistochemical staining for S100 protein, synaptophysin, and/or chromogranin A. Ganglioneuroma lacks the epithelioid component, while GP usually shows a prominent epithelioid component. Paraganglioma lacks ganglion-like cells, and it is very rare in the duodenum [73]. Although NET G1 is the most important differential diagnosis, GP has often been misdiagnosed as NET G1 owing to its low cell proliferative activity. In fact, positive immunoreactivity for neuroendocrine markers and neither mitosis nor prominent Ki-67 immunoreactivity in GP are similar to the findings in NET G1. Nevertheless, as a benign course is more common in cases of GP than in cases of NET G1, it is important to clearly differentiate GP from NET G1. In our multi-institutional retrospective study, we found that the typical epithelioid cells of GP exhibited positive immunoreactivity for the progesterone receptor and pancreatic polypeptide, whereas tumor cells of NET G1 were negative for both markers [6]. Therefore, immunohistochemical analysis of the progesterone receptor and pancreatic polypeptide can assist in differentiating GP from NET G1, even with biopsy specimens.

## 10. Clinical Management of GP

Although GP has been considered an extremely benign neuroendocrine tumor, a previous report mentioned that a patient died from GP [11] and our literature survey found that gastrointestinal bleeding, which can cause severe anemia, was the most common symptom. Furthermore, 25 of 230 cases of GP showed lymph node metastasis and 1 patient has been reported as showing a recurrence due to a residue of a previous tumor at his initial surgical intervention [46]. These findings indicate that surgical or endoscopic intervention for GP is needed and observation with careful follow-up may be undesirable.

In the literature survey, we found that 26 patients underwent an endoscopic procedure for treatment, while only 1 patient required additional surgical intervention owing to the presence of a tumor residue following the initial procedure [47]. These findings indicate that endoscopic procedures can yield favorable results in patients with duodenal GP, if indicated.

Although 2 patients received irradiation after surgical interventions [11, 60], we believe that patients without residual tumors do not require adjuvant therapy because no recurrence or metastasis has been reported in such patients. However, it is still unclear whether a residual tumor can be controlled with irradiation or chemotherapy alone without surgical intervention. Further evaluation of the incidence of GP is warranted. GP has been regarded as an extremely rare NET; however, we previously found that 4 of 10 patients (40.0%) with duodenal NET G1 actually had GP [6]. This suggests that GP accounts for a substantial, constant percentage of duodenal NETs.

## 11. Conclusion

Our new and larger literature survey provided up-to-date clinicopathological information of GP. Since the first case report published by Dahl et al. [8] in 1957, 254 cases of GP have been reported. Our previous literature survey [7] published in 2011 found 192 cases of GP. Therefore, 62 new cases have been identified in the past 5 years, indicating that clinicians and/or pathologists are gradually recognizing the existence of GP and that GP accounts for a substantial percentage of duodenal NETs. As the prognosis differs between GP and NET G1, it is important to differentiate between them. We emphasize the usefulness of immunohistochemical analysis of the progesterone receptor and pancreatic polypeptide for differentiating GP from NET G1, and we believe that this can help improve the clinical management of GP. We found that 1 patient died from GP [11] and that 25 and 3 patients with GP showed lymph node and liver metastases, respectively. To elucidate the risk factors for metastasis and tumor progression of GP, further investigations on GP are required.

## Figures and Tables

**Table 1:** Immunohistochemical findings in each tumor component of gangliocytic paraganglioma.

Immunohistochemical marker	Epithelioid cells	Spindle-shaped cells	Ganglion-like cells
Bcl-2	16.7% (2/12)	58.33% (7/12)	36.36% (4/11)
Calcitonin	20.8% (5/24)	20.0% (4/20)	21.05% (4/19)
CD34	0% (0/2)	33.33% (1/3)	0% (0/1)
CD56	100% (27/27)	50% (9/18)	100% (19/19)
Chromogranin A	74.8% (98/131)	8.8% (9/102)	28.9% (28/97)
Cytokeratins	58.8% (50/85)	6.0% (4/67)	3.0% (2/66)
C-kit	0% (0/8)	0% (0/8)	16.7% (4/11)
Corticotropin	0% (0/0)	0% (0/3)	0% (0/2)
Estrogen receptor	23.1% (3/13)	0% (0/12)	0% (0/11)
Gastrin	4.5% (3/67)	0% (0/62)	0% (0/60)
Glucagon	6.1% (3/49)	0% (0/44)	2.3% (1/43)
Insulin	4.3% (2/47)	0% (0/42)	0% (0/41)
Neurofilament	21.7% (15/69)	69.1% (47/68)	27.3% (18/66)
Neuron-specific enolase	95.7% (90/94)	85.0% (68/80)	87.0% (80/92)
p53	0% (0/13)	0% (0/12)	0% (0/11)
Progesterone receptor	93.3% (14/15)	0% (0/12)	0% (0/12)
Pancreatic polypeptide	91.5% (86/94)	0% (0/83)	32.1% (27/84)
S-100	10.2% (13/128)	96.6% (144/149)	23.9% (28/117)
Serotonin	20.0% (12/60)	1.5% (1/66)	16.7% (9/54)
Somatostatin	82.6% (76/92)	8.7% (6/69)	51.3% (41/80)
Synaptophysin	96.2% (76/79)	54.4% (31/57)	98.4% (61/62)
Vimentin	37.5% (3/8)	60.0% (3/5)	25.0% (1/4)
Vasoactive intestinal peptide	12.1% (4/33)	13.3% (4/30)	10.3% (3/29)

The positive or negative rates in the extracted cases of duodenal gangliocytic paraganglioma are summarized. Listing in alphabetical number.

**Table 2:** Comparison of clinicopathological data between patients with and those without lymph node metastasis.

	Patients with lymph node metastasis	Patients without lymph node metastasis	Statistical analysis
Number of patients	25	205	
Age (years), range	16–74 (median, 50.0)	15–84 (median, 54.0)	No significant difference was found (Mann–Whitney *U* test, *P* = 0.105)
Tumor size (mm), range	10–90 (median, 30.0)	5.5–100 (median, 20.0)	Significant difference was found (Mann–Whitney *U* test, *P* = 0.009)
Sex (male : female)	13 : 12	125 : 78 (2 not reported)	No significant difference was found (*χ* ^2^ test, *P* = 0.390)

A significant difference was found for the tumor size between patients with and those without lymph node metastasis (Mann–Whitney *U* test, *P* = 0.009). This fact means that the size of tumor lesions was significantly larger in patients with lymph node metastasis than in those without lymph node metastasis.

**Table 3:** Comparison of clinicopathological data between patients with gangliocytic paraganglioma (GP) within and those with GP exceeding the submucosa or sphincter of Oddi layer.

	GP within the submucosa or sphincter of Oddi layer	GP exceeding the submucosa or sphincter of Oddi layer	Statistical analysis
Number of patients	81	72	
Age (years), range	16–84 (median, 50.0)	15–73 (median, 57.0)	Significant difference was found (Mann–Whitney *U* test, *P* = 0.016)
Tumor size (mm), range	5.5–65 (median, 21.0)	8–100 (median, 20.0)	No significant difference was found (Mann–Whitney *U* test, *P* = 0.175)
Sex (male to female)	55 : 26	36 : 36	Significant difference was found (*χ* ^2^ test, *P* = 0.032)
Rate of lymph node metastasis	7.4% (6/81)	20.8% (15/72)	Significant difference was found (*χ* ^2^ test, *P* = 0.019)

Significant differences were found for age, sex, and the rate of lymph node metastasis between patients with GP within the submucosa or sphincter of Oddi layer and those with GP exceeding the submucosa or sphincter of Oddi layer.

**Table 4:** Comparison of rate of lymph node metastasis in patients with gangliocytic paraganglioma focusing on tumor size (30 mm).

	Tumor size is within 30 mm	Tumor size is larger than 30 mm
Total number of patients	156	29
Number of patients with lymph node metastasis	16	9
Number of patients without lymph node metastasis	140	20
Rate of lymph node metastasis	10.3% (16/156)	31.0% (9/29)
Statistical analysis	Significant difference was found	
	(*χ* ^2^ test, *P* = 0.019)	

Significant difference was found between them (*χ* ^2^ test, *P* = 0.006). This fact indicated that GP larger than 30 mm is significant risk factor of lymph node metastasis in gangliocytic paraganglioma.
